# Supplementary material for: A Humanized Yeast Phenomic Model of Deoxycytidine Kinase to Predict Genetic Buffering of Nucleoside Analog Cytotoxicity
Source: Genes (Basel). 2019 Sep 30;10(10):770. doi: 10.3390/genes10100770 (PMC6826991; doi:10.3390/genes10100770)
Supplement: Supplementary file 1 [file genes-10-00770-s001.zip › Additional_Files/Additional_File7_GOTermHeatmaps.zip!/Additional_File7_GOTermHeatmaps/mediator_complex.pdf]

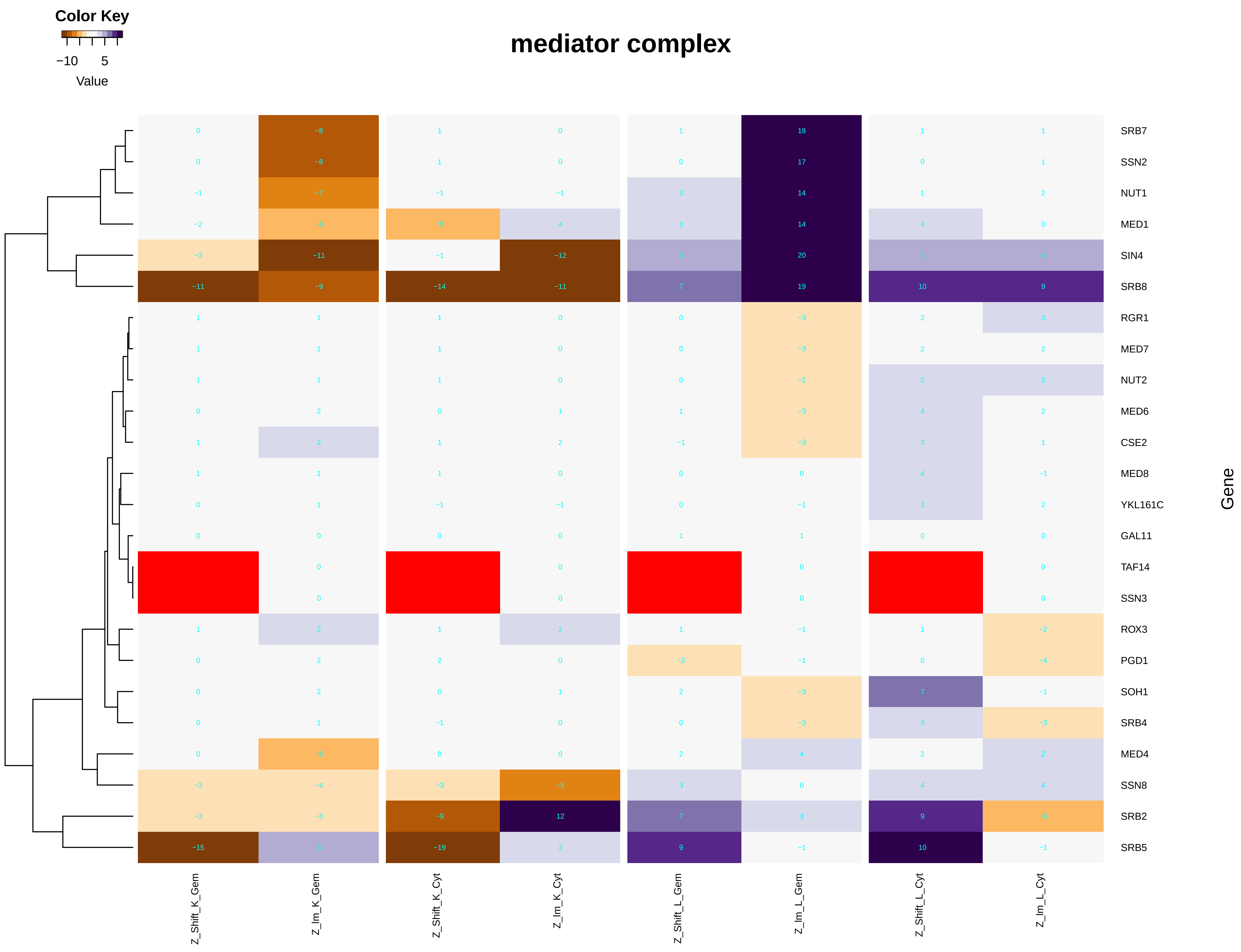
mediator complex
Color Key
−10
5
Value
SRB7
SSN2
NUT1
MED1
SIN4
SRB8
RGR1
MED7
NUT2
MED6
CSE2
MED8
YKL161C
GAL11
TAF14
SSN3
ROX3
PGD1
SOH1
SRB4
MED4
SSN8
SRB2
SRB5
Gene
Z_Shift_K_Gem
Z_Im_K_Gem
Z_Shift_K_Cyt
Z_Im_K_Cyt
Z_Shift_L_Gem
Z_Im_L_Gem
Z_Shift_L_Cyt
Z_Im_L_Cyt
